# Iodide-induced Hyperthyroidism Within Four Hours of Iodide Load: A Case Report and Review

**DOI:** 10.7759/cureus.5960

**Published:** 2019-10-22

**Authors:** Noel D Torres Acosta, Suguni Loku Galappaththy, Brandon Barthel

**Affiliations:** 1 Internal Medicine, University of Missouri-Kansas City School of Medicine, Truman Medical Center, Kansas City, USA; 2 Internal Medicine, University of Missouri-Kansas City School of Medicine, Kansas City, USA; 3 Endocrinology, University of Missouri-Kansas City School of Medicine, Kansas City, USA

**Keywords:** iodide-induced hyperthyroidism, jod-basedow phenomenon, thyroid nodule

## Abstract

The recommended amount of iodide consumption for the majority of adults is approximately 150 mcg per day. During a computed tomography scan, patients can receive 14 to 35 million mcg of iodinated contrast. A 46-year-old African female with no known prior thyroid disease presented with dyspnea and tachycardia. She underwent computed tomography angiogram of the chest to rule out pulmonary embolism. She had evidence of hyperthyroidism four hours after receiving iodinated contrast. We presumed that her hyperthyroidism was a consequence of the Jod-Basedow phenomenon due to an underlying multinodular goiter that was later discovered.

## Introduction

Iodide is an essential component of thyroid hormone synthesis. The recommended amount of iodide consumption for the majority of adults is approximately 150 mcg per day which is typically the average daily intake in the United States [1,2]. Adults can tolerate up to 600-1000 mcg per day without developing side effects [2]. In contrast, there are an estimated 2 billion people with iodide deficiency in the world and 50 million with clinical manifestations [2]. Intravenous iodide-based contrast has on average 14 to 35 million mcg of organic iodide per 100 mL depending on the concentration, which is several times higher than the recommended daily intake [3]. Exposure to large amounts of iodide is managed by the inhibition of iodide organification, which in turn diminishes the synthesis of thyroxine (T4) and triiodothyronine (T3) and increases thyroid-stimulating hormone (TSH) levels, a process known as the Wolff-Chaikoff effect [4]. On the other hand, individuals with certain thyroid pathologies when exposed to large amounts of iodide develop iodide-induced hyperthyroidism (IIH) or the Jod-Basedow phenomenon.

## Case presentation

A 46-year-old Swahili speaking African female presented to the emergency department with complaints of sudden shortness of air (SOA) for three days. SOA worsened with exertion and was associated with headache, back pain, and vague chest discomfort. She had a past medical history of hypertension treated with lisinopril and propranolol. She had emigrated to the United States within the last two years.

On physical examination, her vital signs were as follows: oral temperature, 98.4 Fahrenheit; heart rate, 98 beats per minute (bpm); respiratory rate, 28 breaths per minute; and blood pressure, 158/97 mmHg. Neck examination did not show lymphadenopathy or thyromegaly but she complained of pain on palpation in the submandibular area. Cardiac examination showed rhythmic heart sounds, regular and without murmur; lungs were clear to auscultation, but she had labored breathing and tachypnea. Abdominal examination showed mild tenderness on palpation of the right upper quadrant, no distention, and regular bowel sounds. The rest of the examination was unremarkable.

**Figure 1:**
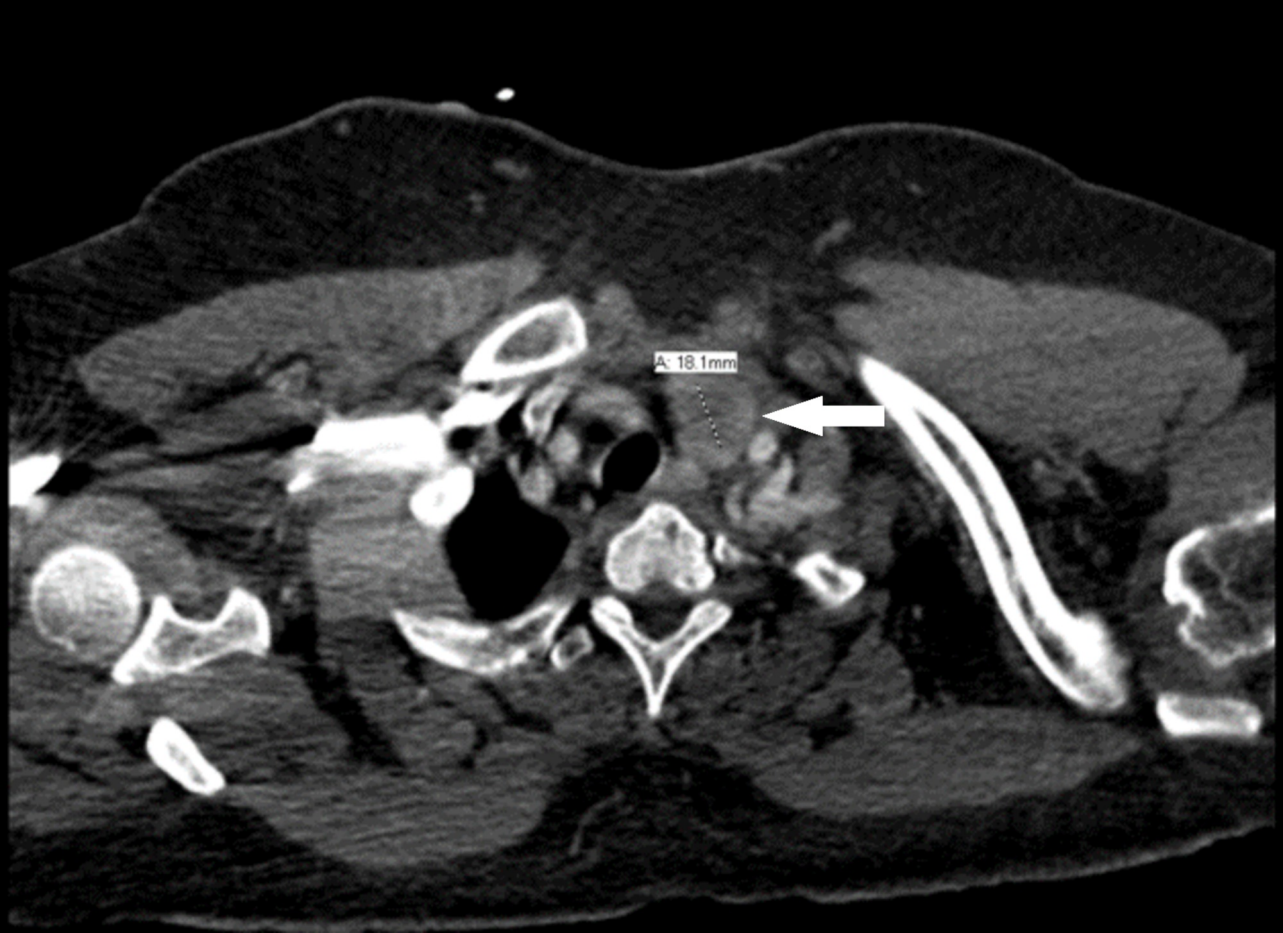
Computed tomography angiogram of the chest showing thyroid nodule

A computed tomography angiogram (CTA) of the chest (Omnipaque 350, 100 mL IV contrast = 35 million mcg of organic iodide) ordered in the emergency department was negative for pulmonary embolism, but it showed an incidental 1.8 cm left thyroid nodule (Figure 1). Four hours later when she was evaluated by the admitting team, TSH level ordered was 0.40 uIU/mL. No free triiodothyronine (FT3) and free thyroxine (FT4) levels were ordered at this time, but they were added later (Table 1). Hematology workup was relevant for a white blood cell count of 16.6 x 10^3^/cmm.

**Table 1:** Thyroid function tests during hospitalization and follow-up. *Values four hours after CT angiogram completion. **Date methimazole and prednisone were started. ***Date methimazole and prednisone were stopped. TSH, thyroid-stimulating hormone; FT3, free triiodothyronine; FT4, free thyroxine.

Test (reference range)	FT3 (2.3-4.2 pg/mL)	FT4 (0.6-1.60 ng/dL)	TSH (0.34-5.60 uIU/mL)
4/17/2019*	17	3.03	0.42
4/18/2019**	12.4	2.67	0.27
4/19/2019	3.1	1.75	
4/20/2019***	2.1	1.43	
4/21/2019	1.9	1.3	
4/22/2019	1.6	1.2	
4/25/2019	2.4	1.2	
6/6/2019		1.08	2.13

**Figure 2:**
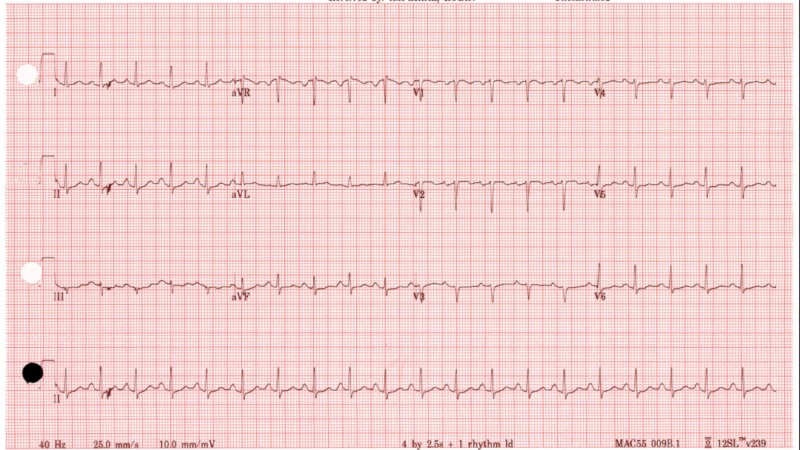
Electrocardiogram showing sinus tachycardia

Later that night, the patient became more tachycardic with a heart rate of 110 bpm and respiratory rate of 30 breaths per minute. Electrocardiogram done at the time showed sinus tachycardia (Figure 2). TSH, FT3, and FT4 levels were ordered with the following results: 0.27 uIU/mL, 12.4 pg/mL, and 2.67 ng/mL, respectively. It was decided to start the patient on methimazole and prednisone, uptitrate propranolol dose, and test for thyroid-stimulating immunoglobin (TSI) and thyroid peroxidase (TPO) antibodies due to suspicion of thyroid storm. Endocrinology was consulted the next day, and it was decided to stop methimazole and prednisone two days after initiation as it was thought that hyperthyroidism was precipitated by iodine contrast load received during CTA. FT3 and FT4 levels were followed during the remainder of her hospitalization with normalization of values (Table 1). TSI and TPO antibodies were normal, supporting the hypothesis of IIH. Thyroid ultrasound showed a single right thyroid nodule measuring 6 mm and two left thyroid nodules measuring 15 and 19 mm, respectively. There was no increased vascularity of the thyroid, which would be suggestive of Graves’ disease. Thyroid scintigraphy was not pursued as the patient had received methimazole recently.

Further investigation for patient’s leukocytosis revealed a peritonsillar abscess. Once antibiotic therapy was initiated, the patient gradually returned to normal.

The patient had repeat thyroid function testing nine weeks after the initial presentation with normalization of TSH and FT4, confirming the diagnosis of transient hyperthyroidism consistent with the Jod-Basedow phenomenon/IIH.

## Discussion

The first description of IIH was reported by Coindet in 1820 on a group of patients receiving 250 mg of iodide for the treatment of goiter [5]. Recently with the increased use of CTA and angiography as diagnostic tools, patients are frequently exposed to supraphysiologic doses of iodide. The reported incidence of contrast-induced hyperthyroidism varies widely from 0.05% to 5% [6]. Our patient received >200,000 times the daily recommended iodide dose with her CTA. Traditionally, the Jod-Basedow phenomenon has been described in patients with underlying thyroid disease, especially in conditions associated with thyroid autonomy. The majority of the cases comprised of patients with euthyroid Graves’ disease, multinodular goiter, residence in an iodide-deficient area, and in the elderly [7]. This patient is originally from an area of endemic iodide deficiency and later discovered to have multinodular goiter. In areas of endemic goiter, the incidence of IIH has been estimated to be up to 1.7% [6]. Even in patients with no prior history of hypothyroidism, there is a strong association between IIH and iodinated contrast exposure (odds ratio 1.98; 95% confidence interval, 1.08-3.60) [8].

The underlying mechanism of IIH is related to chronic thyroid gland stimulation by TSH. This causes mutations in cells within the follicles that produce large quantities of thyroid hormones when iodide becomes sufficiently available like in cases of iodinated contrast administration [9]. The usual timing between iodinated contrast administration and development of hyperthyroidism varies between sources from patients developing symptoms within a few days up to 16 weeks [1,8] This patient had chemical evidence of hyperthyroidism within four hours of receiving iodinated contrast and developed symptoms of hyperthyroidism within nine hours, which to our knowledge is the shortest interval found in our medical literature review. This is an important reminder that even in iodide-replete areas, we still need to think about IIH when treating patients with risk factors such as a prior residence in an iodide depleted area. Moreover, the case reported by Iakovou as well as this case shows that IIH can develop within hours of receiving contrast with previous evidence showing that this developed in days to weeks. IIH is generally self-limiting and resolves within several weeks to months after cessation of iodide exposure. Beta-blockers can be used to blunt overt manifestations of hyperthyroidism [10]. In some refractory or severe cases of hyperthyroidism, antithyroid medication such as methimazole is required [10]. Radioiodine and surgery are only employed in patients with large multinodular goiters or recurrent Graves' disease after the IIH component is resolved to prevent recurrence [10].

Prevention of iodide-induced thyrotoxicosis is an important consideration given the high number of iodinated contrast medium examinations that are conducted in the current medical practice. This is an important concern especially in the geriatric population for several reasons. The geriatric population has a higher incidence of multinodular goiter, clinical hyperthyroidism is more difficult to detect, and this age group has a higher incidence of cardiovascular comorbidities [9]. Several studies have shown that treatment with perchlorate and a thionamide class drug prior to administration of contrast may prevent the development of hyperthyroidism in high-risk groups such as older individuals and patients with multinodular goiter with autonomy [11,12]. This is not commonly practiced in the United States.

## Conclusions

Briefly, our patient developed IIH four hours after receiving iodide contrast in the setting of underlying multinodular goiter. This is the shortest interval to be reported in the literature. Populations with underlying thyroid disease and from endemically of thyroid deficient areas are at great risk for complications when exposed to large amounts of iodide. Careful consideration needs to be taken into account when exposing high-risk patients to supraphysiologic doses of iodide.
